# Oxidative Chemical Stressors Alter the Physiological State of the Nasal Olfactory Mucosa of Atlantic Salmon

**DOI:** 10.3390/antiox9111144

**Published:** 2020-11-18

**Authors:** Carlo C. Lazado, Vibeke Voldvik, Mette W. Breiland, João Osório, Marianne H. S. Hansen, Aleksei Krasnov

**Affiliations:** 1 Nofima, The Norwegian Institute of Food, Fisheries and Aquaculture Research, 1433 Ås, Norway; vibeke.voldvik@nofima.no (V.V.); joao.osorio96@gmail.com (J.O.); marianne.h.s.hansen@nofima.no (M.H.S.H.); Aleksei.Krasnov@Nofima.no (A.K.); 2 Nofima, The Norwegian Institute of Food, Fisheries and Aquaculture Research, 9019 Tromsø, Norway; Mette.W.Breiland@Nofima.no; 3 CIISA, Faculty of Veterinary Medicine, University of Lisbon, 1300-477 Lisbon, Portugal

**Keywords:** fish, mucosal immunity, nasal immunity, oxidative stress, peroxide

## Abstract

The olfactory organs of fish have vital functions for chemosensory and defence. Though there have been some ground-breaking discoveries of their involvement in immunity against pathogens in recent years, little is known about how they respond to non-infectious agents, such as exogenous oxidants, which fish encounter regularly. To this end, we employed Atlantic salmon (*Salmo salar*) as a model to study the molecular responses at the nasal olfactory mucosa of a teleost fish when challenged with oxidants. Microarray analysis was employed to unravel the transcriptional changes at the nasal olfactory mucosa following two types of in vivo exposure to peracetic acid (PAA), a highly potent oxidative agent commonly used in aquaculture: Trial 1: periodic and low dose (1 ppm, every 3 days over 45 days) to simulate a routine disinfection; and Trial 2: less frequent and high dose (10 ppm for 30 min, every 15 days, 3 times) to mimic a bath treatment. Furthermore, leukocytes from the olfactory organ were isolated and exposed to PAA, as well as to hydrogen peroxide (H_2_O_2_) and acetic acid (AA)—the two other components of PAA trade products—to perform targeted cellular and molecular response profiling. In the first trial, microarrays identified 32 differentially expressed genes (DEG) after a 45-day oxidant exposure. Erythrocyte-specific genes were overly represented and substantially upregulated following exogenous oxidant exposure. In Trial 2, in which a higher dose was administered, 62 DEGs were identified, over 80% of which were significantly upregulated after exposure. Genes involved in immune response, redox balance and stress, maintenance of cellular integrity and extracellular matrix were markedly affected by the oxidant. All chemical stimuli (i.e., PAA, H_2_O_2_, AA) significantly affected the proliferation of nasal leukocytes, with indications of recovery observed in PAA- and H_2_O_2_-exposed cells. The migration of nasal leukocytes was promoted by H_2_O_2_, but not much by PAA and AA. The three chemical oxidative stressors triggered oxidative stress in nasal leukocytes as indicated by an increase in the intracellular reactive oxygen species level. This resulted in the mobilisation of antioxidant defences in the nasal leukocytes as shown by the upregulation of crucial genes for this response network. Though qPCR revealed changes in the expression of selected cytokines and heat shock protein genes following in vitro challenge, the responses were stochastic. The results from the study advance our understanding of the role that the nasal olfactory mucosa plays in host defence, particularly towards oxidative chemical stressors.

## 1. Introduction

Oxidative stress is a physiological state in an organism in which the redox balance is altered, as characterised by an increase in the levels of reactive oxygen species (ROS) but normal or low amounts of antioxidants, which may be due to compromised neutralisation property and/or scavenging potential [1,2]. Fish, like many other organisms, have an extensive repertoire to counteract oxidative stress [2,3]. The integrated antioxidant systems, which include enzymatic and nonenzymatic antioxidants, are at the forefront of blocking the harmful effects of ROS [1]. Redox imbalance associated with oxidative stress promotes genetic instability, changes in gene expression patterns, alterations in cellular signalling cascades/cell metabolism, and disruption of the cell cycle, leading to several pathophysiological conditions [4,5].

Oxidants can be endogenously produced or derived from external sources. Endogenous ROS are produced from molecular oxygen as a result of normal cellular metabolism [1], and ROS are constantly produced in all living cells in which roughly up to 1% of an animal’s total oxygen consumption may be attributed to ROS generation and detoxification [6]. Exogenous ROS may come from various sources, and their impacts on redox status have consequences on cell viability, activation, proliferation, and organ function. Farmed fish encounter an increased flow of exogenous ROS several times during a lifetime, as many husbandry practices employ ROS-generating compounds either as a form of disinfectant or water treatment, or as a chemotherapeutant [7,8,9,10,11]. The antimicrobial activity of ROS towards opportunistic and pathogenic microorganisms underlines their use in providing fish with a favourable rearing environment [12]. Nonetheless, our knowledge of the physiological alterations associated with exogenous ROS, mainly from ROS-generating agents being used in fish farming, is fragmentary.

Mucosal organs of fish are multifunctional; besides their role in defence, they carry a multitude of other physiological functions [13,14]. They are often considered the first line of defence because these structures interact with the water matrix where several biological and chemical challenges present themselves regularly. In recent years, there has been a dramatic development in the study of the physiology and immunology of mucosal surfaces in fish, driven mostly by their warranted importance in maintaining the health of farmed fish [13,15].

The nasal olfactory system plays a role not only in chemoreception but also in immune defence, as it is considered an ancient component of the mucosal immune system of vertebrates [16]. It is a highly specialised sensory organ for the detection and identification of minute quantities of chemicals in the environment [17,18], and because water constantly circulates through the nasal cavities, they are continuously prompted with environmental challenges [19]. The mucosal regions of the fish olfactory lamellae have different cellular elements such as goblet cells, sustentacular cells, olfactory sensory neurons, and, most importantly, a rich assemblage of immune cells [19,20,21]. Vertebrate olfactory sensory neurons rapidly sense chemical stimuli in the environment and transduce signals to the central nervous system [18]. The nasopharynx-associated lymphoid tissue (NALT) protects the teleost olfactory organ from water-borne pathogens, just as for airborne pathogens in terrestrial animals [16]. Several recently published studies have demonstrated how viral and bacterial stimulations affect the immunological repertoire of the nasal mucosa in fish. They reveal a very distinct microenvironment that can mount a localised immunity and, at the same time, influence distant immune functions [14,16,18,19,20,22]. Non-infectious agents such as exogenous oxidants are delivered via water and are expected to pass through the nasal cavity of fish. In mammalian models, oxidative stressors are highly potent modulators of the nasal epithelium, and the interaction could induce morphological and pathological alterations [23,24,25]. However, in fish, the influence of exogenous oxidants on the nasal olfactory mucosa is barely explored, despite its common use.

This study explored the impacts of oxidative chemical stressors on the nasal olfactory mucosa of Atlantic salmon (*Salmo salar*). We employed both in vivo and in vitro strategies to unravel the molecular changes in the nasal olfactory mucosa when challenged with exogenous oxidants relevant in fish farming. In this study, we employed peracetic acid (PAA) as the main oxidant, as it is currently being developed as a chemotherapeutant (i.e., for amoebic gill disease—AGD) and disinfectant in recirculating aquaculture systems for salmon [4,26], and the results here are expected to help underline its potential for use. Both in vivo trials were designed to simulate the prospective use of PAA as either a routine disinfectant (Trial 1) or a treatment for a parasitic infection (Trial 2). In addition, in vitro trials were conceived to understand the physiological state of a specific cell type at the mucosa in response to not only PAA but also hydrogen peroxide (H_2_O_2_) and acetic acid (AA). These two compounds are present in equilibrium with PAA in its trade product.

## 2. Materials and Methods

### 2.1. Oxidant Exposure Experiment

**Figure 1:**
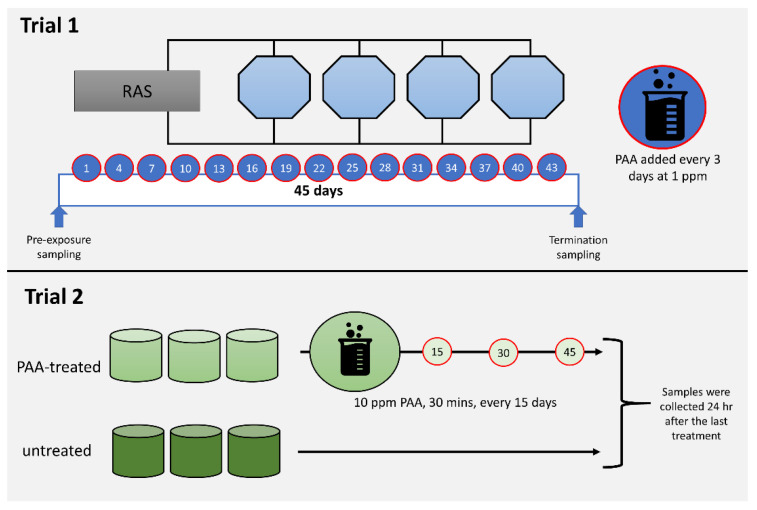
Diagrammatic summary of the in vivo trials. Trial 1 aimed to profile the impacts of periodic and low dose peracetic acid (PAA) application (1 ppm, every 3 days over 45 days), while Trial 2 was designed to investigate the nasal responses following less frequent and high dose PAA treatment (10 ppm for 30 min, every 15 days, 3 times). Details of each trial are described in Section 2.1.

All fish handling procedures described in this paper followed the Guidelines of the European Union (2010/63/EU) and the in vivo exposure trials received approvals from the Norwegian Food Safety Authority (FOTS, Forsøksdyrforvatningen tilsyns- og søknadssystem IDs 20831, 19321). All key personnel have a FELASA (Federation of European Laboratory Animal Science Associations) C certificate to conduct experimentation on live animals. Two independent in vivo trials (Figure 1) were performed in which the application of the exogenous oxidant was based on its proposed use for that particular stage of Atlantic salmon production. It was ensured that all fish used in the experiments (both in vivo and in vitro) did not have a history of oxidant exposure. Peracetic acid is available under different trade products and two commercially available PAA-based disinfectants were used in this study. All three major components of PAA trade (i.e., PAA, hydrogen peroxide and acetic acid) products have disinfection power, though PAA is the most potent contributor to the disinfection property of PAA-based disinfectants.

Trial 1 was conducted at Nofima Centre for Recirculation in Aquaculture (NCRA; Sunndalsøra, Norway) and was aimed at evaluating the impacts of periodic low-dose oxidant exposure on the transcriptome of the nasal olfactory mucosa. This experiment was designed to mimic the use of the oxidant as a routine water disinfectant in a recirculating aquaculture system [27]. Briefly, each of the four 3.2 m^3^ octagonal tanks in a recirculation system was stocked with 735 smolts with an average weight of around 90 g. After four weeks of acclimatisation, the oxidant in the form of a peracetic acid-based disinfectant (Perfectoxid, Novadan ApS, Kolding, Denmark) was directly applied to each tank at a nominal concentration of 1 ppm every 3 days for 45 days, making a total of 15 applications in the duration of the trial. This mode of application was patterned on a previous PAA experiment conducted in rainbow trout, a closely related species of salmon [26]. Moreover, the concentration is within the range safe for use in salmon [4,28]. It was ensured that the application of PAA on each occasion was between 0900 and 1000 to avoid temporal effects. The following parameters were maintained during the trial: water flow rate at 100 L min^−1^, salinity at 11.6 ± 0.5 ‰, temperature at 12.8 ± 0.6 °C, pH at 7.5, dissolved oxygen > 90% saturation, photoperiod at 24 L: 0 D, and a continuous feeding regime (Nutra Olympic 3 mm, Skretting, Averøy, Norway).

Trial 2 was performed at Havbruksstasjonen i Tromsø (HiT; Tromsø, Norway) and was designed to unravel the changes in the nasal transcriptome after repeated but less frequent exposure to higher doses of oxidant. PAA is currently being explored as a potential treatment for amoebic gill disease (AGD), a gill health issue affecting mostly seawater-adapted salmon [4,7]. The trial was designed to simulate an oxidant exposure as a treatment protocol for AGD [26]. Forty fish with an average weight of 80–90 g were stocked into a 500 L circular tank in a flow-through system. There were six tanks in total: three for the control group and three for the oxidant-exposed group. Fish were allowed to acclimatise for a week before the first oxidant treatment was performed. Fish were fasted for 24 h prior to each treatment occasion. Oxidant treatment was performed as follows: Water flow in the tank was stopped. Thereafter, the oxidant (Divosan Forte™, Lilleborg AS, Olso, Norway) was added to the water column to achieve a final concentration of 10 ppm. This concentration was 2x higher than the concentration we earlier tested and reported [4]. Aeration was supplied to enable mixing and to maintain the oxygen level. After 30 min, the water flow was opened, and over 90% of the water was replaced within 10 min. Feeding was continued a day after the exposure. This exposure protocol was performed every 15 days and there were three exposure occasions in the whole trial. The following parameters were maintained during the trial: water flow rate at 6–7 L min^−1^, salinity at 35‰, temperature at 12.0 ± 1 °C, dissolved oxygen > 90% saturation, photoperiod at 24 L: 0 D, and a continuous feeding regime (Nutra Olympic 3 mm, Skretting, Averøy, Norway).

### 2.2. Olfactory Organ Collection

All fish for sampling were humanely euthanised with an overdose of either Tricaine methanesulfonate (Trial 1; MS222, PHARMAQ Ltd., Ås, Norway) or Benzocaine (Trial 2; Benzoak^®^, ACD Pharmaceuticals AS, Oslo, Norway). Percussive stunning was avoided because it triggered the influx of blood to the head, including the olfactory epithelium. Olfactory organ was collected by opening the nostrils to expose the outermost section of the nasal mucosa. The rosette from the left side was then dissected out, immediately suspended in RNA*later*™ (Thermo Fisher Scientific, Sacramento, CA, USA), kept at room temperature overnight for penetration, and then stored at −70 °C until RNA isolation. In Trial 1, olfactory rosettes (*n* = 8 per time-point) were collected before and 45 days (i.e., 24 h after the last PAA application) after the start of periodic oxidant exposure. In Trial 2, the tissue samples (*n* = 9, per group) were collected 24 h after the 3rd exposure.

### 2.3. Isolation of Leukocytes from Olfactory Organ

Leukocytes from olfactory organ were isolated from 15 freshwater-adapted salmon (ca. 80–90 g) with similar genetic background, following a previously published method [22], with slight modifications. Briefly, fish were humanely euthanised with an anaesthetic overdose (Aqui-S, MSD Animal Health, Drammen, Norway). The olfactory organs from both sides were dissected and immediately placed in a modified L-15 (supplemented with 5% foetal bovine serum, 1% Penstrep, 1% HEPES, 4-(2-hydroxyethyl)-1-piperazineethanesulfonic acid) on ice. Rosettes from all fish were combined, and the tissues were cut into small pieces (0.5–1 cm) and mechanically dissociated by incubating the tissue suspension at 4 °C for 30 min with constant agitation. The cell supernatant was collected and temporarily stored at 4 °C. The remaining tissue fragments were suspended in modified L-15 medium and the process of mechanical dissociation was repeated four times. The collected supernatant from the four recurrences was combined and stored at 4 °C. The remaining tissue fragments were suspended in phosphate-buffered saline (PBS) with EDTA (1 mM) and DTT (0.9 mM) and incubated at 4 °C for 30 min with constant gentle agitation. The PBS supernatant was thereafter discarded. Enzymatic digestion was carried out by incubating the remaining fragments in collagenase solution (0.15 mg/mL in L-15, with 1% Penstrep) for 2 h at room temperature (20 °C) with agitation. The supernatants from mechanical dissociations and enzymatic digestion were combined, gently passed through a 100 µm filter, and spun down at 300× *g* for 10 min. The cell pellet was washed and resuspended in modified L-15, laid over a 34%/51% isotonic Percoll^®^ (Sigma-Aldrich, Oslo, Norway) gradient. The tubes were then centrifuged for 30 min at 400× *g* at 4 °C. The cell layer between the gradients was carefully transferred to a tube with modified L-15 medium, centrifuged for 10 min at 400× *g* at 4 °C, and suspended in new modified L-15 medium. Cell viability and number were determined by CellCountess™ II (Thermo Fisher Scientific, Boston, MA, USA). The cells were seeded out onto a 12-well plate (Corning^®^ CellBIND^®^ Surface, Sigma-Aldrich, Oslo, Norway) at a density of 2 × 10^5^ cells per well and incubated at 13 °C.

### 2.4. In Vitro Exposure Trial

The cells were allowed to adhere for 48 h before the exposure was performed. The leukocytes were exposed to three chemical stressors at physiological concentrations—100 µM PAA, 100 µM H_2_O_2_, and 100 µM AA—for 30 min. The concentrations were based on several preliminary in vitro trials and the concentrations were selected because they were able to trigger significant increase in intracellular ROS, thereby providing an indication that the internal redox balance had been altered by the tested oxidants. Each treatment group had four independent wells. Untreated cells served as controls and were handled similarly to the treatment groups, though no chemical stressor was added. After 30 min, the media were removed, cells were washed gently with modified L-15, and 300 µL of the same media was added to each well. After 24 h, the media were removed. The cells were suspended in lysis buffer (ZYMO Quick-RNA™ Microprep kit, Sacramento, CA, USA) and scraped, and the cell suspension was stored at 70 °C until RNA isolation.

### 2.5. Proliferation and Migration Assays

Nasal leukocytes were isolated from 12 freshwater-adapted salmon (ca. 80–90 g) following the method described in Section 2.3 and seeded onto a 96-well plate (Corning^®^, CellBIND^®^ Surface, Sigma-Aldrich, Oslo, Norway) at a density of 10^5^ cells per well. After the cells were allowed to settle and adhere for 24 h, they were exposed to PAA, H_2_O_2_, and AA at a physiological concentration of 100 µM for 30 min and washed. New media were added and the exposed cells were allowed to recover in the incubator. Unexposed cells, serving as controls, were likewise washed, and new media were added. Cell proliferation (proxy for cytotoxicity) was measured using the CyQUANT Direct Proliferation Assay (Thermo Fisher Scientific, Boston, MA, USA) 24 and 48 h after the challenge. Each treatment group, including the unexposed control group, had six replicate wells. Rate of proliferation was expressed relative to the control group of that time-point.

The effects of the chemical stressors on cellular migration were determined by the CytoSelect™ Cell Migration Assay kit (Cell Biolabs, Inc., Sacramento, CA, USA). Freshly isolated nasal leukocytes were suspended in modified L-15 medium without serum. The lower receptacle of the migration chamber was added with L-15 media containing either PAA, H_2_O_2_, or AA in a final concentration of 100 µM. Wells with L-15 medium containing 10% FBS served as the positive control for chemotaxis while L-15 medium alone was designated as a negative control. Each treatment, including the controls, had been assigned three wells. Thereafter, the cells were added to the upper receptacle of the migration chamber at a density of 2 × 10^5^ cells. The migration chamber was incubated for 24 h at 13 °C before the migratory cells were dislodged from the membrane, lysed and labelled with CyQuant^®^ GR dye solution (Sacramento, CA, USA), and fluorescence was read at 480 nm/520 nm.

### 2.6. Intracellular ROS Quantification

Nasal leukocytes were isolated, cultured, and treated with the chemical stressors as described in detail in Section 2.4 and Section 2.5. The intracellular level of reactive oxygen species (ROS) in the treated cells, including untreated control, was quantified using the OxiSelect™ Intracellular ROS Assay Kit (Cell Biolabs, Inc., San Diego, CA, USA) at 24 and 48 h after challenge. The level of ROS is given as a proportion of fluorescent dichlorodihydrofluorescein (DCF).

### 2.7. RNA Isolation, cDNA Synthesis, and Quantitative Real-Time PCR

The RNA from both olfactory tissues and nasal leukocytes were isolated using Quick-RNA™ Microprep kit (Zymo Research, CA, USA). RNA concentration was measured in a NanoDrop 1000 Spectrophotometer (Thermo Fisher Scientific, DE, USA), and the quality of the samples for microarray was further assessed using an Agilent^®^ 2100 Bioanalyzer™ RNA 6000 Nano kit (Agilent Technology Inc., Santa Clara, CA, USA). All samples had an RNA Integrity Value higher than 8.8.

A High Capacity RNA-to-cDNA Reverse Transcription kit (Applied Biosystems, Sacramento, CA, USA) was used to prepare the complementary DNA from the nasal leukocyte samples using 300 ng RNA input following a synthesis protocol of 25 °C for 10 min, followed by 37 °C for 120 min and then 5 min at 85 °C. The expression of selected antioxidant defence, cytokines, and heat shock protein genes (Supplementary File 1) was quantified using the PowerUp™ SYBR™ Green master chemistry (Applied Biosystems, Sacramento, CA, USA) in a QuantStudio5 real-time quantitative PCR system (Applied Biosystems, Sacramento, CA, USA). The qPCR reaction mixture included 4 µL of diluted cDNA, 5 µL SYBR™ Green Master (Thermo Fisher Scientific, Boston, MA, USA), and 1 µL of the forward and reverse primer. All samples were run in duplicate, including minus reverse transcriptase and no template controls. The thermocycling protocol included pre-incubation at 95 °C for 2 min, amplification with 40 cycles at 95 °C for 1 s and at 60 °C for 30 s, and a dissociation step series of 95 °C for 15 s, 60 °C for 1 min, and 95 °C for 15 s. Amplification efficiency was calculated from a five-point standard curve of 2-fold dilution series of pooled cDNA. The expression of the target genes was normalised using three reference genes, namely *elongation factor 1a* (*eef1a*), *acidic ribosomal protein* (*arp*), and *β-actin* (*actb*) [29].

### 2.8. Microarray Analysis

Olfactory rosettes from Trials 1 and 2 were subjected to microarray analysis using Nofima’s Atlantic salmon DNA oligonucleotide microarray SIQ-6 (custom design, GPL16555, Sacramento, CA, USA), which contains 15 K probes for protein-coding genes involved in immunity, tissue structure, integrity and functions, cell communication and junctions, and extracellular matrix, amongst many others [30]. Agilent Technologies manufactured and supplied the microarrays, reagents, and equipment used in the analysis. Using 110 ng of total RNA template per reaction, RNA was amplified using a One-Color Quick Amp Labeling Kit, and thereafter Cy3 was labelled. Subsequently, fragmentation of the labelled RNA was carried out using a Gene Expression Hybridization Kit and hybridisation followed in an oven thermostatted at 65 °C with a constant rotation speed of 10 rpm for 17 h. The arrays were washed in sequence with Gene Expression Wash Buffers 1 and 2 and were scanned through an Agilent SureScan Microarray scanner. Data processing was carried out in Nofima’s bioinformatics package STARS.

### 2.9. Data Handling and Statistics

The significant difference in the transcript level of the target marker genes between before and after periodic oxidant exposure in Trial 1 and between the unexposed-control and oxidant-exposed groups in Trial 2 was determined by Student’s t-test for independent samples; the threshold of differential expression in microarray analyses was 1.75-fold. The level of significance was set at 5% (*p* < 0.05).

A Shapiro–Wilk test was used to evaluate the normal distribution and a Brown–Forsyth test to check for equal variance of the proliferation assay and gene expression data set. Two-way ANOVA was then employed to investigate significant differences amongst treatment groups over time. In addition, the Holm–Sidak test was used to identify pairwise differences. One-way ANOVA was used for migration assay data. All statistical tests were performed using SigmaPlot 14.0 Statistical Software (Systat Software Inc., London, UK), with a level of significance set at *p* < 0.05.

## 3. Results

### 3.1. Transcriptomic Changes in the Olfactory Rosettes from Trial 1

After 45 days of periodic low-dose oxidant exposure, microarray analysis identified 32 differentially regulated genes (DEG) in the nasal olfactory rosette (Table 1, Supplementary File 2). The numbers of upregulated (16/32) and downregulated genes were equal (16/32). Erythrocyte-specific genes were the most represented group, with nine transcripts/variants identified as being upregulated in response to the exogenous oxidant. Genes involved in immune response such as *c-c motif chemokine 28*, *interleukin 13 receptor alpha-2*, *defensin beta 4*, *ig heavy chain*, and *mannose-specific lectin-like*, were all downregulated after 45 days of oxidant exposure. Two genes encoding cytokeratins were downregulated. Three out of four genes with metabolic functions were likewise downregulated.

### 3.2. Transcriptomic Changes in the Olfactory Rosettes from Trial 2

Sixty-two DEGs were identified in the olfactory rosettes from fish exposed to an oxidant on three occasions, 56 of which, accounting for 82% of the DEGs, were upregulated (Table 2, Supplementary File 2). From this group, genes related to immunity, including cytokines and effectors, were largely represented with 14 upregulated transcripts. Genes with innate immune functions constitute a considerable number in the DEG panel. Genes with known involvement in cellular structural integrity such as *keratin* and *plekstrin* were likewise upregulated. A similar effect was observed on genes encoding extracellular proteins (e.g., *fibronectin*, *mucin5b*). Several genes involved in various metabolic pathways were represented in the DEGs panel, such as those involved in amine, amino acid, calcium, and xenobiotic metabolism. A total of 3/4 DEGs of lipid metabolism were downregulated following oxidant exposure. Exogenous oxidant exposure upregulated the expression of genes with function in cellular processes such as DNA replication, signalling, and protein folding/modification including the *heat shock proteins*. Oxidant-induced changes in cellular redox balance were likewise manifested with two DEGs.

### 3.3. Effects of Oxidative Chemical Stressors on Leukocyte Proliferation and Migration

**Figure 2:**
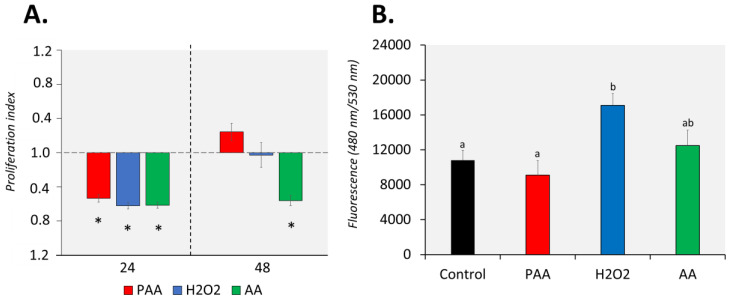
Effects of oxidative chemical stressors on the (**A**) proliferation and (**B**) migration of nasal olfactory leukocytes. For the proliferation assay, cells were isolated and cultured for 2 days before they were treated with 100 µM of PAA (peracetic acid), hydrogen peroxide (H_2_O_2_), and acetic acid (AA) for 30 min. Proliferation was quantified 24 and 48 h after challenge. Asterisk (*) denotes that proliferation was significantly affected relative to the unstimulated group. Results are presented as index of proliferation, where we expressed the proliferation relative to the control, unstimulated group. Two-way ANOVA followed by the Holm–Sidak test identified statistical difference amongst treatment groups over time. For migration assay, cells were allowed to migrate to a chamber containing the chemical stressor at a 100 µM concentration for 24 h. The positive control group has foetal bovine serum (FBS) as a chemoattractant. One-way ANOVA was used for migration assay data. Different letters a,b indicate significant difference at *p* < 0.05. Results are presented as mean ± SD of 5 (proliferation)/3 (migration) wells, with cells from 12 fish.

The proliferation of nasal leukocytes 24 h after the challenge was significantly affected by PAA, as well as by its two main components, H_2_O_2_ and AA (Figure 2A). Cellular proliferation reduced by at least 0.5-fold in all treatment groups and no inter-treatment differences were observed. After 48 h, nasal leukocytes exposed to PAA and H_2_O_2_ slightly recovered, and the proliferation rate did not significantly differ from that of the control group. However, the effect of AA on proliferation was still persistent after 48 h, when the proliferation index in the group was 0.6-fold lower compared to control. Moreover, a significant difference was observed between the AA-exposed and two other treatment groups.

PAA and AA did not significantly affect the migration potential of the nasal leukocytes (Figure 2B). On the other hand, H_2_O_2_ promoted the migration of nasal leukocytes with a significant increase compared to control. A comparison of treatments revealed that H_2_O_2_-induced migration was significantly different from PAA but not from AA.

### 3.4. Level of ROS in Chemically Stressed Cells

**Figure 3:**
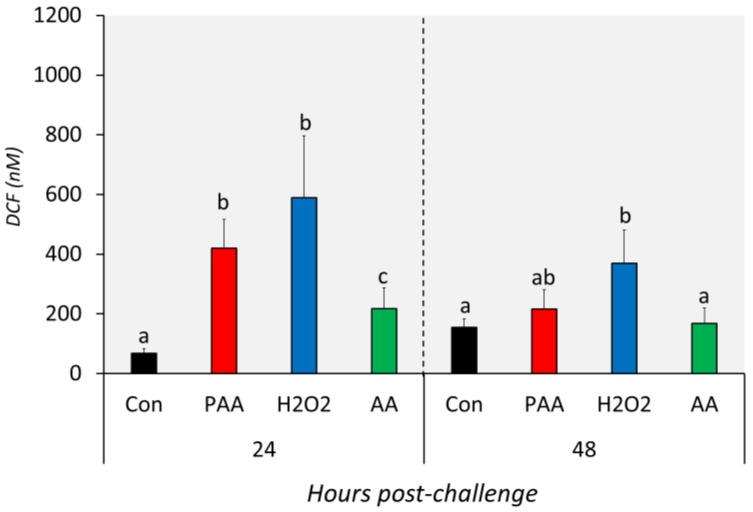
Levels of intracellular reactive oxygen species in nasal olfactory leukocytes exposed to different oxidative chemical stressors. The level was measured at 24 and 48 h after exposure to 100 µM of PAA (peracetic acid), H_2_O_2_, and AA (acetic acid) for 30 min. The control group was handled similarly, but without any chemical stimulation. Two-way ANOVA followed by the Holm–Sidak test identified statistical difference amongst the treatment groups over time. Different letters a,b indicate a significant difference at *p* < 0.05 between groups within a time-point. There was no time-related difference within a treatment group. Results are presented as mean ± SD of 5 wells, with cells from 12 fish.

The three chemical stressors significantly increased the intracellular ROS level of nasal olfactory leukocytes 24 h after challenge (Figure 3). The highest increment was identified in H_2_O_2_-exposed cells, followed by PAA and AA. In addition, the intracellular ROS of PAA- and H_2_O_2_-exposed cells were significantly higher than in the AA-exposed group at this time-point. After 48 h, the ROS level in PAA and AA-exposed cells displayed no significant difference from the control. The intracellular ROS in H_2_O_2_-exposed cells remained elevated 48 h after challenge. Moreover, the level was significantly higher compared to the control and AA-exposed cells but not to the PAA-exposed cells. Though not statistically significant, the level in PAA- and H_2_O_2_-exposed cells was apparently lower than the measured level at 24 h post-challenge.

### 3.5. Changes in the Expression of Antioxidant Defence Genes in the Nasal Olfactory Leukocytes

**Figure 4:**
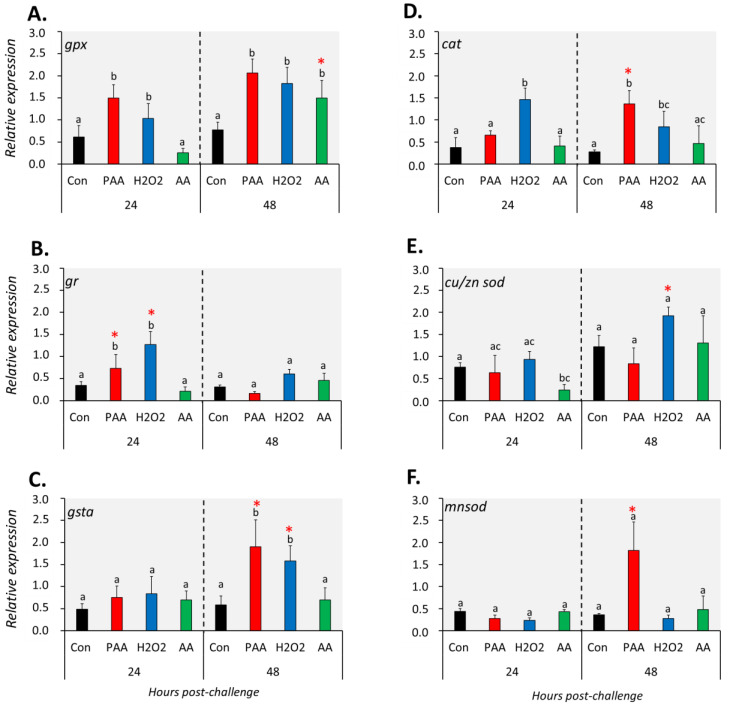
Changes in the expression of antioxidant defence genes following exposure to oxidative chemical stressors (**A**–**F**). The transcript level of six selected genes (i.e., *gpx*, *gr*, *gsta*, *cat*, *cu/zn sod*, *mnsod*) was quantified by RT-qPCR at 24 and 48 h after exposure to 100 µM of PAA, H_2_O_2_, and AA for 30 min. The control group was handled similarly, but without any chemical stimulation. Two-way ANOVA followed by the Holm–Sidak test identified statistical difference amongst treatment groups over time. Different letters indicate a significant difference at *p* < 0.05 amongst the treatment groups within a time-point. Asterisk (*) denotes that the level in a treatment group differs between the two time-points. Results are presented as mean ± SD of four wells, with ca. 10^5^ cells from 15 fish.

The expression of six genes with known function in antioxidant defence was differentially affected by the three chemical stressors (Figure 4). The transcript level of *gpx* was significantly higher in PAA- and H_2_O_2_-exposed cells compared to control at both time-points (Figure 4A). Such an increase was only identified in AA-exposed cells 48 h after challenge and the expression was significantly higher than the level at 24 h post-challenge. Chemically induced stress resulted in quite the opposite temporal expression profiles for *gr* and *gsta*. An elevated level of *gr* transcripts was observed in PAA- and H_2_O_2_-exposed groups at 24 h post-challenge while *gsta* expression was significantly altered after 48 h (Figure 4B,C). In both cases, the expression relative to the other time-point was significantly higher. The transcription of both genes in AA-exposed cells remained unaltered at both time-points. *Cat* expression was significantly upregulated in H_2_O_2_-exposed cells at both time-points, whereas for the PAA-exposed group, a significant increase was observed only 48 h post-challenge (Figure 4D). There was also a significant difference in *cat* expression in the PAA-exposed group between the two time-points. AA did not elicit a significant transcriptional change from *cat*. *Cu/zn sod* was significantly downregulated in the AA-exposed group as compared to control at 24 h, but not at 48 h after challenge. While *cu/zn sod* expression was unaltered in the PAA-exposed group at both time-points, a significant increase was observed in the H_2_O_2_-exposed group but only after 48 h. *Cu/zn sod* expression in H_2_O_2_-exposed cells at 24 h was likewise higher compared to the level at 48 h. The overall expression of *mnsod* displayed no significant alterations, except in PAA-exposed cells 48 h after challenge, where the expression was at least three times higher than the control and other treatment groups.

### 3.6. Changes in the Gene Expression of Cytokine and Heat Shock Proteins in the Nasal Olfactory Leukocytes

**Figure 5:**
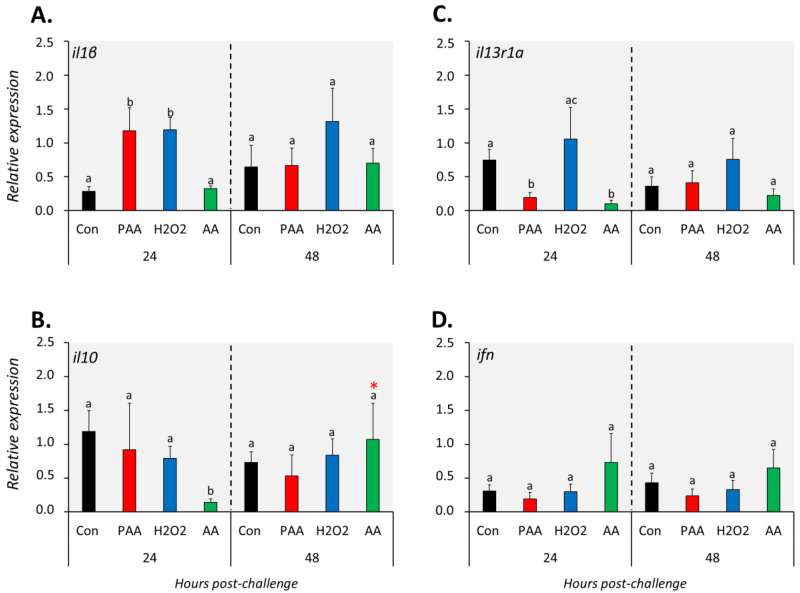
Changes in the expression of cytokine genes following exposure to oxidative chemical stressors (**A**–**D**). The transcript level of 4 selected genes (i.e., *il1β*, *il10*, *il13r1a*, *ifn*) was quantified by RT-qPCR at 24 and 48 h after exposure to 100 µM of PAA, H_2_O_2_, and AA for 30 min. The control group was handled similarly, but without any chemical stimulation. For the explanation on statistics and notations, please refer to Figure 4.

The expression of *il1β* increased significantly in PAA- and H_2_O_2_-exposed groups compared to controls 24 h after challenge, but such a change was no longer observed at 48 h (Figure 5A). On the other hand, AA exposure did not alter *il1β* expression at both time-points. Generally, *il10* expression was not significantly affected by the oxidative chemical stressors, except in AA-exposed cells at 24 h post-challenge, where a significant downregulation was observed. The transcript level of *il13r1a* was significantly lower in PAA- and AA-exposed cells compared to control 24 h after challenge, though the change did not persist until 48 h (Figure 5C). The expression of *ifn* was unaltered in most treatment scenarios at both time-points (Figure 5D).

**Figure 6:**
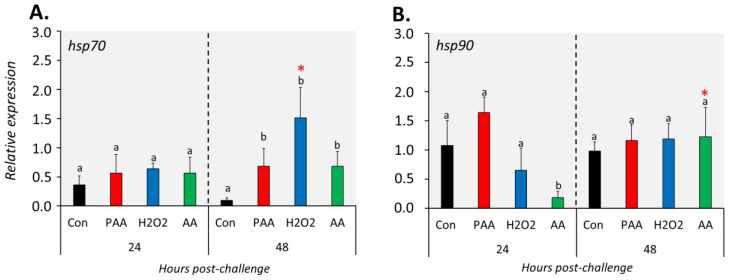
Changes in the expression of gene coding for heat shock proteins following oxidative chemical challenge (**A**,**B**). The transcript level of two selected genes (i.e., *hsp70*, *hsp90*) was quantified by RT-qPCR at 24 and 48 h after exposure to 100 µM of PAA, H_2_O_2_, and AA for 30 min. The control group was handled similarly, but without any chemical stimulation. For the explanation on statistics and notations, please refer to Figure 4.

The three chemical stressors did not significantly change the expression of *hsp70* 24 h post-challenge (Figure 6A). After 48 h, however, *hsp70* expression was significantly elevated in all treatment groups compared to control, and the highest increment was identified in H_2_O_2_-exposed cells. *Hsp90* expression was not significantly altered in PAA- and H_2_O_2_-exposed cells at both time-points (Figure 6B). A significant downregulation was detected in AA-exposed cells at 24 but not at 48 h post-challenge, and the expression was higher in the latter than with the former time-point.

## 4. Discussion

The present study reveals, through in vivo and in vitro exposure trials, how the nasal olfactory mucosa of Atlantic salmon mobilised its defence repertoires when challenged with oxidative chemical stressors. To our knowledge, this is the first report that demonstrates the molecular changes initiated by chemically induced oxidative stress in the nasal mucosa of a teleost fish. Using a nasal leukocyte model, it was further shown how the constituent oxidants of the tested therapeutics alter the cellular redox balance and the associated response mounted by a specific group of cells at the nasal olfactory mucosa to these challenges.

The two in vivo exposure studies uncovered the molecular repertoire of the nasal olfactory mucosa when challenged with either a periodic-low dose or less frequent-high dose of the oxidative agent PAA. In the first trial, we addressed the nasal consequences in the application of oxidant as a routine disinfectant in salmon. No conclusive implications can be drawn as to whether the exogenous oxidant was a stimulator or an inhibitor of nasal mucosal physiology as the ratios of upregulated and downregulated genes were equal after 45 days of exposure. However, two groups—haemoglobins and immune genes—displayed a trend in response to the exogenous oxidant. Haemoglobin (Hb) is a predominant component in erythrocytes responsible for oxygen transport to the different tissues in vertebrates [31]. The Hb transcripts were upregulated in the nasal mucosa following periodic low-dose oxidant exposure, and both α and β subunits were represented. In murine models, it has been demonstrated that overexpression of Hb affected a network of genes involved in O_2_ homeostasis, which subsequently resulted in the suppression of oxidative stress [32,33]. Exposing HepG2 cells to H_2_O_2_ induced the expression of both haemoglobin α and β, and their overexpression likewise resulted in cellular protection against oxidative stress [34]. The upregulation of several haemoglobin transcripts in response to periodic low-dose oxidant challenge was probably a protective mechanism of the nasal mucosa against oxidative stress. Despite the limited number, there was an indication that intermittent oxidant exposure may negatively regulate nasal immunity. The constitutive presence of oxidant in the environment may trigger several responses from an organism – continual mounting of protective action, accommodation, or habituation (i.e., decreasing response through time). It was reported earlier that periodic application of oxidant (i.e., PAA) in trout—a species closely related to salmon – somehow resulted in a dampening response, which could be indicative of habituation [27,35]. Such a consequence was likewise implicated in salmon post-smolts [28]. This partly sheds light on the downregulation of these immune genes after several weeks of exposure. We cannot disregard the potential oxidant-mediated immunosuppression in the nasal mucosa, as some of these transcripts have earlier been implicated in compromised immunity under oxidative stress in higher animal models [36,37].

Trial 2 provided a relatively clearer picture of how an oxidant administered at a higher dose, but less frequently, altered the nasal transcriptome as shown with a higher number of DEGs and a prominent regulatory profile. It was evident that oxidant treatment resulted in dysregulation of nasal redox balance, and hence triggered mucosal oxidative stress. In earlier publications, we have demonstrated that antioxidant defences in mucosal tissues (i.e., gills and skin) were remarkably altered by a similar oxidant though delivered at a much lower concentration [4,7]. The mobilisation of these antioxidant defences following oxidant exposure highlighted the capability of mucosal surfaces to muster physiological responses to increased environmental ROS, thereby protecting the mucosa from eventual oxidative damage, as supported by in vitro cell works demonstrated here as well. A substantial upregulation in immune-related genes was also observed, which likely offered insights into the complementarity of the immune and antioxidant defence mechanism at the mucosa during oxidative stress. There is a tight relationship between oxidative stress and immunity, and often the co-regulation of these two defence mechanisms provides robust responses during oxidative challenges [3,38]. One of the immune effector molecules that were markedly regulated was *ornithine decarboxylase*, a gene coding for an enzyme responsible for catalysing the conversion of ornithine to putrescine, the first and rate-limiting step in the synthesis of putrescine and the polyamines spermidine and spermine [39]. It is important to highlight that *spermidine/spermine N1-acetyltransferase 1*, a key molecule in amine metabolism, and with a counteractive function against oxidative stress, was significantly upregulated too. Increased expression of *ornithine decarboxylase* had been demonstrated in human hepatoma HUH7 cells subjected to chemically induced oxidative stress [40]. The upregulation observed in the present study is indicative of a similar function for protection against oxidative damage. Heat shock proteins play a role in several cellular processes that occur during and after exposure to oxidative stress [41]. There are indications, both in this trial as well as from previous studies using this oxidant, that it can induce transient oxidative stress [4,7,27,28]. The upregulation of both *hsps* in the nasal mucosa following oxidant exposure suggests intervention in oxidative stress-triggered changes by correction of conformation or selecting and directing aberrant proteins to the proteasome or lysosomes for degradation, in which these molecules are known to be key regulators. Besides the activation of several effector molecules that provide an interconnected response to oxidant-triggered oxidative stress, it is compelling to observe that several genes for molecules for cytostructural and extracellular matrix were represented in the list of upregulated genes. Previous studies using this oxidant found histostructural changes, though minimal, in the gill and skin mucosa [4,27,42]. We can speculate that the upregulation observed here may play a role in maintaining the structural integrity and barrier functionality under oxidative challenge. The upregulation of two genes responsible for extracellular matrix, *GMP giant mucus protein* and *mucin 5b*, implies that mucus physiology is affected by the oxidant and that the changes in these two genes underline their function in providing a layer of protective defence at the mucosa. Two related genes have been shown to participate in modulating the mucus layer of the olfactory epithelium in mammalian models [43,44], and they likely exert a similar function in the nasal mucosa of salmon. However, this must be functionally ascertained in the future. DEGs with known functions in lipid metabolism were downregulated in the oxidant-challenged nasal mucosa. Though it is difficult to conclude whether oxidative stress triggered an imbalanced lipid metabolism in the nasal mucosa because of a limited panel of DEGs under this category, it is interesting to note that such interaction has been reported in other animals [45,46]. The downregulation observed in these lipid metabolic mediators indicates that oxidant exposure may interfere with this process, though the magnitude remains an open question.

We then focused on one specific cell type at the nasal mucosa to investigate how the cells respond to the oxidant, as well as to the two other constituents of PAA trade products. The proliferation of nasal olfactory leukocytes was affected by the three chemical stressors 24 h after exposure at an almost similar rate. However, such an influence was no longer observable 48 h after exposure in PAA- and H_2_O_2_-exposed cells. This indicates that the effects on cellular proliferation following PAA and H_2_O_2_ stimulation could be transitory, and the cells were able to recover quickly. AA was more potent in inhibiting cellular proliferation, as the effects persisted until 48 h. Nasal olfactory leukocytes exhibited migratory potential, as all factors resulted in migration of the cells, though at varying levels. Cell migration plays an important role in many normal biological and pathophysiological processes, and oxidants can either promote or inhibit migration [47]. H_2_O_2_ administered individually and not in mixture with PAA and AA modulated the migration of the nasal olfactory leukocytes, suggesting its potent chemoattractant function, as demonstrated by earlier studies in other animal models [47,48]. The migratory potential of nasal olfactory leukocytes is vital in orchestrating a cellular response when environmental ROS levels reach a concerning concentration or when pathophysiological response had been caused by oxidative stress.

We further asked: *If the oxidants triggered changes in the cellular phenotypic response* (i.e., proliferation and migration), *can they also induce oxidative stress*? The oxidant used had been shown to prompt oxidative stress at the systemic [7] and tissue [4,7] levels, but this has yet to be demonstrated at the cellular level. Exposure to the three chemical oxidative stressors resulted in an increase in the intracellular concentration of ROS, which indicates that it induced redox imbalance; hence, oxidative stress occurred. Both H_2_O_2_ and AA have long been identified as inducers of oxidative stress [11,48,49]. Here, we have shown that PAA and H_2_O_2_ were far more potent inducers of intracellular ROS production than AA in nasal leukocytes. Interestingly, for both PAA and AA, induction of intracellular level was transient because, after an elevated level 24 h after exposure, the concentration returned to the normal/control level. This was not observed in H_2_O_2_-exposed cells; their intracellular ROS was still at an elevated level 48 h after exposure, though the level was slightly lower compared to that at 24 h. This indicates that cells exposed to PAA and AA have a faster capability to abate an increased intracellular ROS level than H_2_O_2_-exposed cells, which further suggests that H_2_O_2_ has a higher and more persisting impact as an oxidative stressor on the nasal leukocytes. Different oxidants could trigger different mechanisms of oxidative stress induction [1,50], and somehow the observations in the present study are in agreement with this differential regulatory impact.

The increased intracellular ROS incited by the three chemical stressors initiated a response from the antioxidant system of the nasal olfactory leukocytes. The expression of enzymatic antioxidants was predominantly upregulated, indicating their key role towards the threats of the oxidative stressors. The overall expression profile of these antioxidant genes shows that PAA and H_2_O_2_ were more potent triggers than AA, which, to some extent, is in agreement with the results in terms of how the oxidative stressors affected the intracellular ROS level. Antioxidant markers *gpx*, *gr*, *gsta*, and *cat* were perhaps the key response molecules, as their expression was significantly elevated in at least one time-point in PAA- and H_2_O_2_-exposed cells. The response profile can be divided into two arbitrary groups based on their elevated temporal expression – early (i.e., *gr*) and late (i.e., *gsta* and *cat*) oxidant responders. *Glutathione peroxidase* metabolises H_2_O_2_ to H_2_O, and the reduced glutathione provides an antioxidant function by resetting the redox status in tissues [51]. *Gpx* has been implicated in the responses of fish to environmental toxicants that can trigger oxidative stress [52], and this mechanism may also be working in nasal leukocytes. We have previously documented oxidant-induced alteration in *gpx* expression in the gills and skin of salmon; hence, the results provide further evidence that it is a vital molecule for the mucosal antioxidant system in this fish species [7]. It is interesting to note that *glutathione reductase gr* was upregulated both in the cells and as one of the DEGs identified in Trial 2. *Gr*, as an antioxidant, is responsible for the regeneration of reduced glutathione during detoxification of peroxides and free radicals formed in mitochondria, thus maintaining the redox potential of the cell [53]. The upregulation of *gr* in both instances, as well as in previous oxidant studies in salmon [7,10], provides strong support for its canonical function in mucosal antioxidant defence, that is likely ubiquitously regulated by the oxidative chemical stressor. There was no apparent tendency for AA-induced changes in the antioxidant repertoire in the nasal olfactory leukocytes, though both *gpx* and *cu/zn sod* were responsive.

The overall profile in the expression of cytokine markers and heat shock protein genes could not be conclusively established, though the stochastic changes provide insights into how oxidative stressors may likely influence these molecules. The relationship between oxidants and inflammatory response is well-established in mammalian systems [54], though such interaction is less understood in fish, especially concerning exogenous oxidative stressors. We have shown that *il1b* expression was modulated by PAA and H_2_O_2_ 24 h after exposure. An earlier publication reported that increased IL-1β stimulated glutathione production, thereby protecting neurons from oxidative damage [55]. The present data could not decisively ascertain whether such a directional effect was also initiated in the nasal leukocytes; however, the upregulation of *il1b* and the two genes of glutathione metabolism offer a potential link. In Trial 2, we have identified several cytokines genes that were upregulated following the oxidant challenge. It is possible that this increase in expression facilitated the migration of inflammatory cells to the epithelial surface where the oxidant was in close contact. One of the areas that must be explored in the future is the early phase of the inflammatory response, which was not captured by the current data. Both *hsp70* and *hsp90* were significantly upregulated in the olfactory rosette in Trial 2, but a similar change in the cell experiment could not be observed. The mode of oxidant application may play in part in this apparent difference.

## 5. Conclusions

Fish encounter environmental oxidants during production, as several husbandry practices rely on the use of oxidative chemical compounds, such as during water disinfection [27] or disease treatment [9]. Application frequency may vary, from continuous to periodic, each depending on its intended use (i.e., disinfectant vs. therapy). PAA is a potent oxidant, though the window of safe dose is limited [56]. The present study contributes to a better understanding of how the nasal olfactory mucosa of Atlantic salmon, one of the least explored mucosal tissues with regards to redox physiology, mount physiological and immunological responses when prompted with exogenously generated oxidative challenges. Oxidative stress is a physiological imbalance that requires a coordinated response to protect the organism from oxidative damage and, eventually, facilitate repair and recovery. The nasal mucosa of salmon can activate different molecules that may likely participate in the adaptive responses to oxidative stress. Nasal immunology is one of the emerging fields in fish immunology research [20], and the several oxidant-responsive genes identified in the paper are potential molecules for in-depth functional characterisation for their role in the nasal microenvironment, mainly towards non-infectious agents. One area that is interesting for future experiment is on whether exogenous antioxidants (e.g., in-feed antioxidants) can mitigate the effects of the chemical oxidative stressors by augmenting the innate antioxidant system of fish.

## Figures and Tables

**Table 1:** Some of the differentially expressed genes in the olfactory rosette of salmon from Trial 1. Transcripts are annotated for their known or predicted function. Expression data are ratios of means of 45 days after the exposure to pre-exposure.

Annotation	Name	Fold
Cytoskeleton	*Keratin, type I cytoskeletal 20*	−2.69
Cytoskeleton	*Keratin, type I cytoskeletal*	−3.16
DNA replication	*DNA replication licensing factor MCM6*	2.34
Chemokine	*C-C motif chemokine 28*	−4.33
Cytokine receptor	*Interleukin 13 receptor alpha-2*	−1.83
Antibacterial	*Defensin beta 4*	−2.60
B cell	*Ig heavy chain*	−3.69
Lectin	*Mannose-specific lectin-like*	−2.20
Lipid metabolism	*Phosphoethanolamine/phosphocholine phosphatase*	−2.43
Protease	*Duodenase-1*	−2.20
Protease	*Serine protease 23-like*	1.76
Energy metabolism	*Pyruvate dehydrogenase kinase isozyme 2*	−2.12
Tissue ECM mucus	*Mucin-2*	2.14
Erythrocyte	*Hemoglobin subunit beta-1*	2.58
Erythrocyte	*Hemoglobin subunit alpha (5)*	2.80
Erythrocyte	*Hemoglobin subunit alpha-4-like (2)*	5.40
Erythrocyte	*Hemoglobin subunit beta*	1.76

For genes with several variants (number enclosed in parentheses), mean values are presented. NB: The complete list of differentially expressed genes (DEGs) is given in Supplementary File 2.

**Table 2:** Some of the differentially expressed genes in the olfactory rosette of salmon from Trial 2 are annotated for their known or predicted function. Expression data are ratios of means of the 10 ppm PAA-treated group to the unexposed/control treated group. Samples were collected 24 h after the 3rd exposure.

Annotation	Name	Fold
Cytoskeleton	*Keratin 14*	1.83
Cytoskeleton	*Keratin 4*	2.05
Cytoskeleton	*Keratin cytoskeletal 17*	3.48
Cytoskeleton	*Pleckstrin 2*	2.11
DNA replication	*DNA replication licensing factor MCM6*	1.75
Protein folding	*14-3-3 protein alpha*	1.82
Protein folding	*Heat shock cognate 70 (2)*	3.63
Protein folding	*Heat shock protein 90, alpha (2)*	3.53
Signaling	*Guanine nucleotide binding protein*	2.06
Redox homeostasis	*Glutathione reductase, mitochondrial*	2.26
Redox homeostasis	*Redox-regulatory protein FAM213A*	−2.67
Signaling	*Tyrosine phosphatase type IVA, member 1 (2)*	1.97
Cell Surface	*Vacuole membrane protein 1*	2.12
B cell	*IgH-locus*	1.90
Cytokine receptor	*Interferon-alpha/beta receptor alpha chain*	1.93
Cytokine receptor	*Interleukin 13 receptor alpha-2*	1.96
Cytokine receptor	*Interleukin-1 receptor type II (2)*	2.37
Effector	*Differentially regulated trout protein 1*	2.85
Effector	*Ornithine decarboxylase (3)*	2.28
Effector	*Thrombin-like enzyme cerastocytin*	2.85
Lymphocyte	*T-lymphocite maturation associated protein*	2.72
T cell	*CD276 antigen-like*	2.66
TNF	*TNF decoy receptor*	1.90
Effector	*Spermidine/spermine N1-acetyltransferase 1*	4.07
Amino acid metabolism	*Methionine adenosyltransferase II, alpha b*	1.97
Calcium metabolism	*Protein S100-A1*	2.22
Iron metabolism	*Ferritin, heavy polypeptide 1b (5)*	2.34
Lipid metabolism	*Mid1-interacting protein 1-like*	−1.93
Lipid metabolism	*Phospholipid transfer protein*	−1.92
Lipid metabolism	*Short-chain dehydrogenase/reductase 3*	−2.18
Mitochondria	*Malic enzyme 3, NADP*	−1.87
Protease	*Calpain 9 (2)*	2.24
Steroid metabolism	*Cholesterol 25-hydroxylase-like protein A*	1.92
Sugar metabolism	*Glycogen debranching enzyme*	−1.90
Xenobiotic metabolism	*Glutathione S-transferase P-like*	2.44
Tissue ECM	*Fibronection*	1.74
Collagen	*Collagen alpha-2(VI) chain*	−3.01
Mucus	*GMP Giant mucus protein*	1.85
Mucus	*Mucin-5B (2)*	1.91
Mucus	*Arylalkylamine N-acetyltransferase*	−1.92
Epithelium	*Epithelial membrane protein 2-like*	1.75
Secretory	*Gastrotropin-like*	2.83
Lipid metabolism	*Globoside alpha-1,3-N-acetylgalactosaminyltransferase 1-like*	2.62

For genes with several variants (number enclosed in parentheses), mean values are presented. NB: The complete list of DEGs is given in Supplementary File 2.

## Supplementary Materials

The following are available online at https://www.mdpi.com/2076-3921/9/11/1144/s1, Supplementary File 1. List of primers used in qPCR analysis. Supplementary File 2. List of differentially expressed genes identified in Trials 1,2.
