# Supplementary material for: Oxidative Chemical Stressors Alter the Physiological State of the Nasal Olfactory Mucosa of Atlantic Salmon
Source: Antioxidants (Basel). 2020 Nov 18;9(11):1144. doi: 10.3390/antiox9111144 (PMC7698894; doi:10.3390/antiox9111144)
Supplement: Supplementary file 1 [file antioxidants-09-01144-s001.zip › Lazado et al. Oxidant-Olfactory 2020 Supplementary File 1.pdf]

Supplementary File 1. Primers used in the present study.

Gene name	Abbreviation	Num.	Sequence (5' – 3')	Reference
Glutathione peroxidase	*gpx*	BG934453	F: GATTCGTTCCAAACTTCCTGCTA<br>R: GCTCCCAGAACAGCCTGTTG	[1]
Glutathione reductase	*gr*	BG934480	F: CCAGTGATGGCTTTTTTGAACTT<br>R: CCGGCCCCCACTATGAC	[1]
Glutathione S-transferase	*gsta*	NM_001141492	F: AGGGCACAAGTCTAAAGAAGTC<br>R: GTCTCCGTGTTTGAAAGCAG	[2]
Manganese superoxide dismutase	*mnsod*	DY718412	F: GTTTCTCTCCAGCCTGCTCTAAG<br>R: CCGCTCTCCTTGTCGAAGC	[1]
Copper/Zinc superoxide dismutase	*cu/znsod*	BG936553	F: CCACGTCCATGCCTTTGG<br>R: TCAGCTGCTGCAGTCACGTT	[1]
Catalase	*cat*	BG935638	F: GGGCAACTGGGACCTTACTG<br>R: GCATGGCGTCCCTGATAAA	[3]
Interleukin 1β	*il1b*	AY617117	F: AGGACAAGGACCTGCTCAACT<br>R: CCGACTCCAACTCCAACACTA	[4]
Interleukin 10	*il10*	EF165029	F: GGGTGTCACGCTATGGACAG<br>R: TGTTTCCGATGGAGTCGATG	[4]
Interleukin 13 receptor 1 alpha	*il13ra*	KY432362	F: ACCAATCAGGTCCCAGAAGA<br>R: GATGAACCGTTGAGAGTCCC	[5]
Interferon c	*ifnc*	NM_001279097.1	F: ATGTATGATGGGCAGTGTGG<br>R: CCAGGCGCAGTAACTGAAAT	[6]
Heat shock protein 70	*hsp70*	BG933934	F: CCCCTGTCCCTGGGTATTG<br>R: CACCAGGCTGGTTGTCTGAGT	[1]
Heat shock protein 90	*hsp90*	contig03769	F: CCACCATGGGCTACATGATG<br>R: CCTTCACCGCCTTGTCATTC	[3]
Elongation factor alpha-1	*ef1a*	BG933853	F: GAATCGGCTATGCCTGGTGAC<br>R: GGATGATGACCTGAGCGGTG	[7]
Β-actin	*actb*	XM_014194537	F: CCAAAGCCAACAGGGAGAA<br>R: AGGGACAACACTGCCTGGAT	[8]
Acidic ribosomal protein	*arp*	XM_014167939	F: TCATCCAATTGCTGGATGACTATC<br>R: CTTCCCACGCAAGGACAGA	[8]

1. Solberg, M.F.; Kvamme, B.O.; Nilsen, F.; Glover, K.A. Effects of environmental stress on mRNA expression levels of seven genes related to oxidative stress and growth in Atlantic salmon Salmo salar L. of farmed, hybrid and wild origin. *BMC Res Notes* **2012**, *5*, 672-672, doi:10.1186/1756-0500-5-672.
2. Lazado, C.C.; Voldvik, V. Temporal control of responses to chemically induced oxidative stress in the gill mucosa of Atlantic salmon (Salmo salar). *Journal of Photochemistry and Photobiology B: Biology* **2020**, 111851.
3. Olsvik, P.A.; Vikeså, V.; Lie, K.K.; Hevrøy, E.M. Transcriptional responses to temperature and low oxygen stress in Atlantic salmon studied with next-generation sequencing technology. *BMC Genomics* **2013**, *14*, 817, doi:10.1186/1471-2164-14-817.
4. Ingerslev, H.-C.; Rønneseth, A.; Pettersen, E.F.; Wergeland, H.I. Differential Expression of Immune Genes in Atlantic Salmon (Salmo salar L.) Challenged Intraperitoneally or by Cohabitation with IPNV. *Scandinavian Journal of Immunology* **2009**, *69*, 90-98, doi:10.1111/j.1365-3083.2008.02201.x.
5. Sequeida, A.; Castillo, A.; Cordero, N.; Wong, V.; Montero, R.; Vergara, C.; Valenzuela, B.; Vargas, D.; Valdés, N.; Morales, J., et al. The Atlantic salmon interleukin 4/13 receptor family: Structure, tissue distribution and modulation of gene expression. *Fish & Shellfish Immunology* **2020**, *98*, 773-787, doi:https://doi.org/10.1016/j.fsi.2019.11.030.
6. Jenberie, S.; Thim, H.L.; Sunyer, J.O.; Skjødt, K.; Jensen, I.; Jørgensen, J.B. Profiling Atlantic salmon B cell populations: CpG-mediated TLR-ligation enhances IgM secretion and modulates immune gene expression. *Scientific Reports* **2018**, *8*, 3565, doi:10.1038/s41598-018-21895-9.
7. Garcia de la serrana, D.; Johnston, I.A. Expression of Heat Shock Protein (Hsp90) Paralogues Is Regulated by Amino Acids in Skeletal Muscle of Atlantic Salmon. *PLoS One* **2013**, *8*, e74295, doi:10.1371/journal.pone.0074295.
8. Sanden, M.; Olsvik, P.A. Intestinal cellular localization of PCNA protein and CYP1A mRNA in Atlantic salmon Salmo salar L. exposed to a model toxicant. *BMC Physiology* **2009**, *9*, 3, doi:10.1186/1472-6793-9-3.
