# Supplementary material for: Oxidative Chemical Stressors Alter the Physiological State of the Nasal Olfactory Mucosa of Atlantic Salmon
Source: Antioxidants (Basel). 2020 Nov 18;9(11):1144. doi: 10.3390/antiox9111144 (PMC7698894; doi:10.3390/antiox9111144)
Supplement: Supplementary file 1 [file antioxidants-09-01144-s001.zip › Lazado et al. Oxidant-Olfactory 2020 Supplementary File 2.pdf]

Annotation	Name	Fold
Cytoskeleton	Keratin 14	1.83
Cytoskeleton	Keratin 4	2.05
Cytoskeleton	Keratin cytoskeletal 17	3.48
Cytoskeleton	Pleckstrin 2	2.11
DNA replication	DNA replication licensing factor MCM6	1.75
Protein folding	14-3-3 protein alpha	1.82
Protein folding	Heat shock cognate 70	4.56
Protein folding	Heat shock cognate 70	2.70
Protein folding	Heat shock protein 90, alpha	5.02
Protein folding	Heat shock protein 90, alpha	2.04
Signaling	Guanine nucleotide binding protein	2.06
Redox homeostasis	Glutathione reductase, mitochondrial	2.26
Redox homeostasis	Redox-regulatory protein FAM213A	-2.67
Signaling	Tyrosine phosphatase type IVA, member 1	1.96
Signaling	Tyrosine phosphatase type IVA, member 1	1.98
Cell Surface	Vacuole membrane protein 1	2.12
B cell	IgH-locus	1.90
Cytokine receptor	Interferon-alpha/beta receptor alpha chai	1.93
Cytokine receptor	Interleukin 13 receptor alpha-2	1.96
Cytokine receptor	Interleukin-1 receptor type II	2.36
Cytokine receptor	Interleukin-1 receptor type II	2.38
Effector	Differentially regulated trout protein 1	2.85
Effector	Ornithine decarboxylase	1.97
Effector	Ornithine decarboxylase	2.25
Effector	Ornithine decarboxylase	2.62
Effector	Thrombin-like enzyme cerastocytin	2.85
Lymphocyte	T-lymphocite maturation associated prote	2.72
T cell	CD276 antigen-like	2.66
TNF	TNF decoy receptor	1.90
Effector	Spermidine/spermine N1-acetyltransferas	4.07
Amino acid metabolism	Methionine adenosyltransferase II, alpha b	1.97
Calcium metabolism	Protein S100-A1	2.22
Iron metabolism	Ferritin, heavy polypeptide 1b	2.30
Iron metabolism	Ferritin, heavy polypeptide 1b	2.36
Iron metabolism	Ferritin, heavy polypeptide 1b	2.48
Iron metabolism	Ferritin, heavy polypeptide 1b	2.20
Iron metabolism	Ferritin, heavy polypeptide 1b	2.34
Lipid metabolism	Mid1-interacting protein 1-like	-1.93
Lipid metabolism	Phospholipid transfer protein	-1.92
Lipid metabolism	Short-chain dehydrogenase/reductase 3	-2.18
Mitochondria	Malic enzyme 3, NADP	-1.87
Protease	Calpain 9	2.22
Protease	Calpain 9	2.26
Steroid metabolism	Cholesterol 25-hydroxylase-like protein A	1.92
Sugar metabolism	Glycogen debranching enzyme	-1.90
Xenobiotic metabolism	Glutathione S-transferase P-like	2.44
Tissue ECM	Fibronection	1.74
Collagen	Collagen alpha-2(VI) chain	-3.01
Mucus	GMP Giant mucus protein	1.85
Mucus	Mucin-5B	1.85
Mucus	Mucin-5B	1.97
Mucus	Arylalkylamine N-acetyltransferase	-1.92
Epithelium	Epithelial membrane protein 2-like	1.75
Secretory	Gastrotropin-like	2.83
Lipid metabolism	Globoside alpha-1,3-N-acetylgalactosam	2.62
Unknown Uncertain	uroplakin-3b-like protein	2.01

Unknown Uncertain	sarcolemmal membrane-associated prote	-1.94
Unknown Uncertain	Integral membrane protein 2A	-1.77
RNA metabolism	Y box binding protein 1	1.73
Hematopoiesis	Transmembrane 4 L6 family member 1	4.06
Neural	Tetraspanin-3	2.04
Differentiation	Secreted frizzled-related protein 1	-1.79
